# Aflatoxin B_1_-Induced Developmental and DNA Damage in *Caenorhabditis elegans*

**DOI:** 10.3390/toxins9010009

**Published:** 2016-12-26

**Authors:** Wei-Hong Feng, Kathy S. Xue, Lili Tang, Phillip L. Williams, Jia-Sheng Wang

**Affiliations:** 1 Department of Environmental Health Science, College of Public Health, University of Georgia, Athens, GA 30602, USA; sollis308@hotmail.com (W.-H.F.); ksxue@uga.edu (K.S.X.); pwilliam@uga.edu (P.L.W.); jswang@uga.edu (J.-S.W.); 2 Wuxi Center for Disease Control and Prevention, Wuxi 214023, Jiangsu, China

**Keywords:** Aflatoxin B_1_, apoptosis, *Caenorhabditis elegans*, DNA damage response, growth and reproductive toxicity

## Abstract

Aflatoxin B_1_ (AFB_1_) is a ubiquitous mycotoxin produced by toxicogenic *Aspergillus* species. AFB_1_ has been reported to cause serious adverse health effects, such as cancers and abnormal development and reproduction, in animals and humans. AFB_1_ is also a potent genotoxic mutagen that causes DNA damage in vitro and in vivo. However, the link between DNA damage and abnormal development and reproduction is unclear. To address this issue, we examined the DNA damage, germline apoptosis, growth, and reproductive toxicity following exposure to AFB_1_, using *Caenorhabditis elegans* as a study model. Results found that AFB_1_ induced DNA damage and germline apoptosis, and significantly inhibited growth and reproduction of the nematodes in a concentration-dependent manner. Exposure to AFB_1_ inhibited growth or reproduction more potently in the DNA repair-deficient *xpa-1* nematodes than the wild-type N2 strain. According to the relative expression level of pathway-related genes measured by real-time PCR, the DNA damage response (DDR) pathway was found to be associated with AFB_1_-induced germline apoptosis, which further played an essential role in the dysfunction of growth and reproduction in *C. elegans*.

## 1. Introduction

Aflatoxins are the most studied group of mycotoxins produced by *Aspergillus flavus* and *Aspergillus parasitics*, which naturally present in a variety of animal feeds and human foods, particularly in cereal grains, nuts, and feedstuffs [1]. Aflatoxin B_1_ (AFB_1_) is the most toxic and hepatocarcinogenic among the known mycotoxins [2]. Although the liver is clearly the principal target organ for AFB_1_, considerable evidence suggests that the toxin also induces lung and colorectal cancers [3,4]. 

AFB_1_ is mainly metabolized by cytochrome P450 enzymes into the genotoxic metabolite, 8,9-epoxide-AFB_1_ (AFBO). AFBO can bind to DNA and cause the formation of AFB_1_-DNA adducts [5]. AFB_1_-DNA adducts may block normal replication and transcription, thereby causing lesions in the double-stranded DNA. Normally, the DNA lesions can be repaired or removed by the nucleotide excision repair system (NER) [6]. If the lesion persists in the genome DNA, it can lead to mutagenesis and carcinogenesis. 

Apart from mutagenic and carcinogenic effects, AFB_1_ can also produce other serious toxic effects, such as growth retardation, immune suppression, and reproductive malfunction in humans and domestic animals [7,8,9]. The mechanism of growth retardation or reproductive malfunction caused by AFB_1_ has not been fully investigated to date.

*Caenorhabditis elegans* has been found to be an excellent model organism for both fundamental assessments and mechanistic studies in the fields of environmental and mechanistic toxicology [10], owing to its easy maintenance, cellular simplicity, genetic manipulability, and evolutionarily-conserved biology. In *C. elegans*, although somatic cells are almost invariant, the germline keeps proliferating through the majority of its life. The *C. elegans* germline is known to undergo apoptosis initiated either by DNA damage agents or non-genotoxic mechanism. Generally, genotoxic exposure activates the DNA damage response (DDR) signaling pathway (also called checkpoint pathway) to induce germline apoptosis, in which checkpoint gene *hus-1* and tumor suppressor gene *p53* (called *cep-1* in *C. elegans*) are essential components [11]. Leung et al. found that AFB_1_ can induce DNA damage in *C. elegans* [10,12]; however, to the best of our knowledge, the germline apoptosis induced by AFB_1_ has not been reported. 

Building on the previous findings, the present study investigated the toxic effects of AFB_1_ on growth and reproduction of *C. elegans*. We also assessed the DNA damage and DNA damage-dependent germline apoptosis caused by AFB_1_, and attempted to explore the underlying mechanisms of AFB_1_-induced growth inhibition and reproduction malfunction.

## 2. Results

### 2.1. AFB_1_ Effects on the Growth of C. elegans

**Figure 1:**
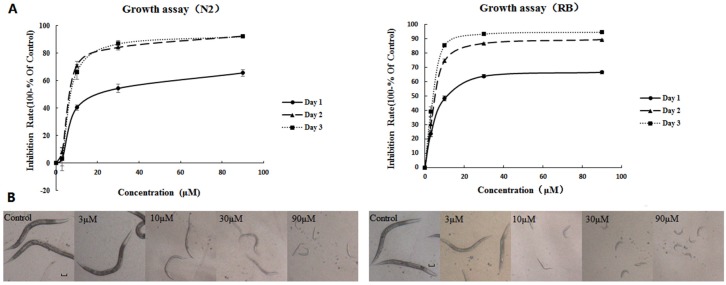
(**A**) Inhibitory effects on growth of *C. elegans* exposed to AFB_1_ with four concentrations (3, 10, 30, and 90 μM) at 24 h, 48 h, and 72 h; and (**B**) morphological changes of *C. elegans* following 72 h exposure to AFB_1_ (scale is 100 μm for all photographs).

Synchronized L1 wild-type (N2) and NER-deficient *xpa-1* strains were exposed to 3, 10, 30, and 90 μM AFB_1_ for three days and body size was measured every 24 h. All controls and 3-μM exposed worms reached adulthood by day 3, while higher-exposed (10, 30, and 90 μM ) nematodes remained in the larvae stage on day 3, as indicated by morphology and the inhibition of egg-laying. AFB_1_ showed significant growth inhibition effects on N2 or *xpa-1* strains in a concentration-dependent manner (*p* < 0.001) as shown in Figure 1. Maximal growth inhibition rates were observed at day 2 for the N2 strain and day 3 for the *xpa-1* strain. Additionally, as indicated by Figure 1, exposure to AFB_1_ resulted in greater growth inhibition in the *xpa-1* strain as compared to the N2 strain. This point could also be corroborated by the EC_50_ values of the two strains (showed in Table 1), and statistical analysis indicated that the EC_50_ values of the *xpa-1* strain was significantly lower than those of the N2 strain (*p* < 0.05). 

### 2.2. AFB_1_ Effects on the Brood Size of C. elegans

**Figure 2:**
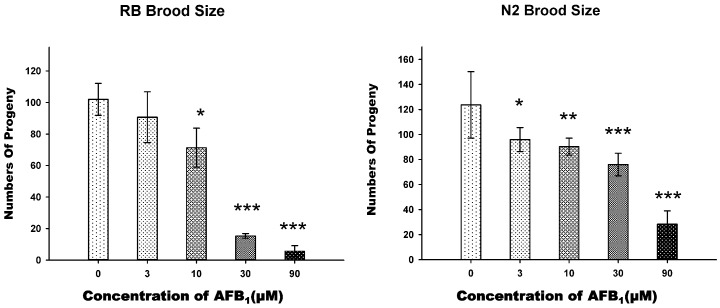
Effects of AFB_1_ on reproduction (brood size) of N2 and RB (*xpa-1*) *C. elegans* after 72 h exposure to AFB_1_. All values are presented as the means ± SD; *** *p* < 0.001; ** *p* < 0.01; * *p* < 0.05, significantly different from the control nematodes.

To determine whether AFB_1_ exposure affected the fertility of *C. elegans*, we measured the brood size of the N2 and *xpa-1* (RB69) young adult hermaphrodites which were exposed individually to graded concentrations of AFB_1_. All of the adults survived for the entire exposure time. Brood size was determined by counting the total number of hatched eggs and, therefore, it was an indicator of both reproduction and embryonic development. In the exposure groups of 10, 30, and 90 μM, both N2 and *xpa-1* strain worms exhibited significant decreases in brood sizes (*p* < 0.001), which were approximately 30% (10 μM), 40% (30 μM), and 80% (90 μM) lower than that of the control group in the N2 strain, while, in the *xpa-1* strain, the percentage decreases were 30% (10 μM), 85% (30 μM), and 95% (90 μM), respectively (Figure 2). In lower-exposure groups (3 and 10 μM), there were no apparent differences in inhibition effects between *xpa-1* and N2 strains. In contrast, in the higher-exposure groups in the *xpa-1* strain, reproduction was significantly more affected by the AFB_1_ exposure (*p* < 0.05) (Figure 2).

### 2.3. DNA Damage Assay

**Figure 3:**
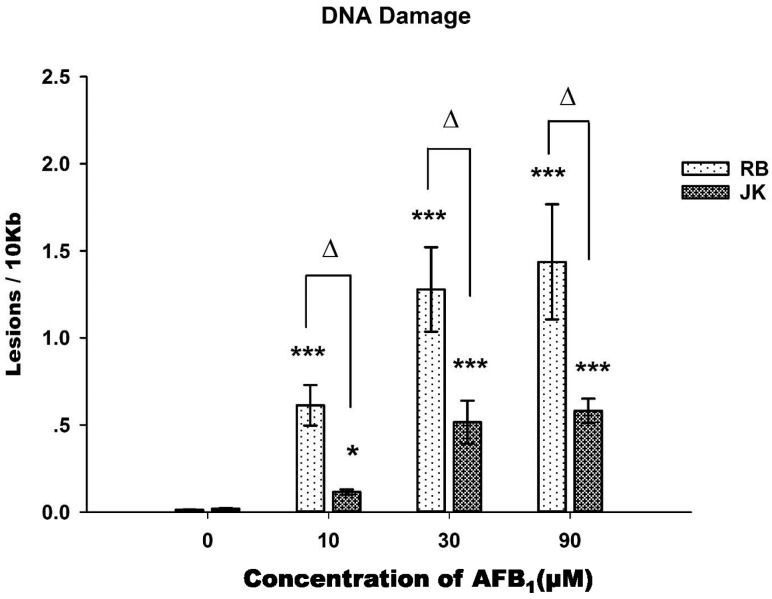
DNA damage in young adult *xpa-1* (RB) and *glp-1* (JK) nematodes following AFB_1_ exposure for 48 h. All values are presented as the means ± SD; *** *p* < 0.001; * *p* < 0.05, significantly different from the control nematodes. Δ *p* < 0.01, significantly different between the nematode strains treated with the same level of AFB_1_.

L4 *glp-1* (JK1107) and *xpa-1* (RB69) strains were exposed to different concentrations of AFB_1_ for 48 h, and DNA damages were measured. The *glp-1* strain (JK1107) is a temperature-sensitive mutant strain, which, when maintained at 25 °C, will block its germline proliferation and, therefore, stops cell division. This eliminates the “diluting effect” of the new DNA produced by dividing germ cells, avoiding interferences when measuring DNA damage [13]. The lesion frequency increased significantly (Figure 3) in the nuclear genomes of the *xpa-1* or *glp-1* strains with the increased concentration of AFB_1_ (*p* < 0.001). The *xpa-1* strain was also found to be more susceptible to AFB_1_ exposure than that of the *glp-1* strain for all treated groups, with DNA lesion frequency in the *xpa-1* strain about two times higher than that of the *glp-1* strain (*p* < 0.01) at higher concentrations. This increased the susceptibility of the DNA repair-deficient *xpa-1* strain, as well as the concentration response in toxicity elicited by AFB_1_, which were consistent with what was found in growth and brood size assays.

### 2.4. AFB_1_-Induced Germ Line Apoptosis

**Figure 4:**
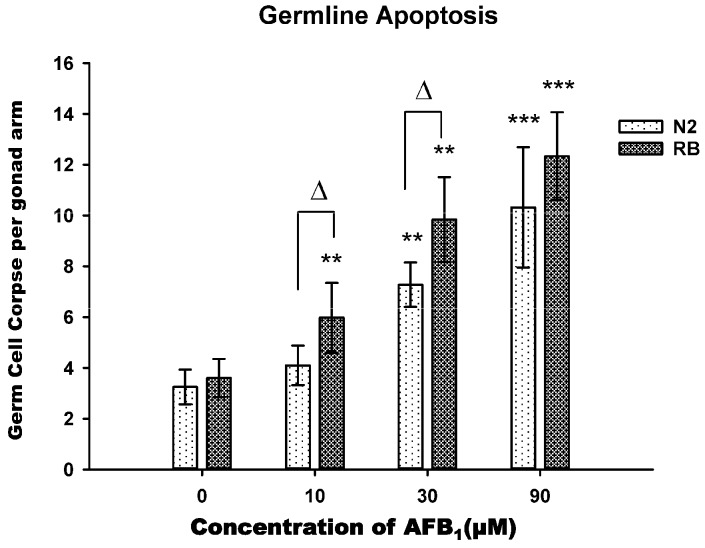
AFB_1_ induced germline apoptosis in *C. elegans*. Synchronized N2 and RB (*xpa-1*) young adults were treated with indicated concentrations of AFB_1_ for 48 h, and germ cell corpses per gonad arm were determined. All values were represented as the mean ± SD. *** *p* < 0.001; ** *p* < 0.01, significantly different from the control nematodes. Δ *p* < 0.05, significantly different between the nematode strains treated with the same level of AFB_1_.

To determine whether AFB_1_ exposure induced germline cell apoptosis of *C. elegans*, synchronized young adult hermaphrodites were exposed to different concentrations of AFB_1_ solutions. The Acridine Orange(AO)-stained nuclei per gonad arm were counted after 48 h of exposure, as shown in Figure 4. There were significant concentration-related increases in apoptosis (*p* < 0.001). At 10 μM, no difference was found between the treated group and the control group for N2 strain, while significant differences were found for *xpa-1* strain (*p* > 0.05). At higher concentrations, however, there was a significant increase in apoptosis cells when treatment concentratons were increased to 30 μM (*p* < 0.01) or 90 μM (*p* < 0.001), especially in *xpa-1* strain (Figure 4). Statistical analysis also found that AFB_1_ was more prone to induce germline apoptosis in the *xpa-1* strain than in the N2 strain (*p* < 0.05) in the 10 and 30 μM exposure groups.

### 2.5. AFB_1_-Induced Gemline Apoptosis via the Checkpoint Pathway, Not ERK Pathway

**Figure 5:**
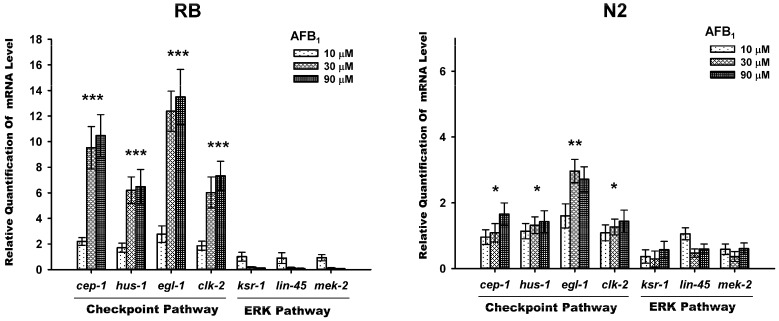
Expression levels of checkpoint pathway and ERK pathway genes after AFB_1_ treatment in L4 wild-type N2 and RB (*xpa-1*) strains following 48 h exposure. The DNA damage checkpoint was activated following AFB_1_ exposure in *C. elegans*; all values were represented as the mean ± SD. *** *p* < 0.001; ** *p* < 0.01; * *p* < 0.05, significantly different from the control nematodes.

The real-time PCR results showed that the expression of genes for checkpoint proteins, *egl-1*, *hus-1*, *clk-2*, and *cep-1* were significantly increased in AFB_1_-exposed N2 and *xpa-1* strains when compared to the untreated nemotodes (*p* < 0.05). Apart from *egl-1*, the increase in the expression of these genes also showed a concentration-dependent manner (Figure 5). The degree of gene inductions in the *xpa-1* strain was about five times higher than that of the N2 strain. The activation of the ERK pathway has been found to promote proliferation, differentiation, survival, and cell apoptosis. To determine the roles of ERK signalings in AFB_1_-induced apoptosis, the expression level of the genes involved in ERK pathway, *lin-45* (MAPKKK), *mek-2* (MAPKK), and *ksr-1* (ku68) were measured. For both the *xpa-1* and N2 strains exposed to AFB_1_, there were no significant differences in the expression levels of *mek-2*, *ksr-1* and *lin-45* as compared to the untreated nemotodes (Figure 5). 

## 3. Discussion

AFB_1_, a potent chemical carcinogen, is mainly metabolized by cytochrome P450 enzymes into a genotoxic metabolic intermediate, AFBO, which can bind to DNA and cause the formation of AFB_1_-DNA adducts. AFB_1_-DNA adducts can be removed or repaired by nucleotide excision repair (NER) in the nuclear genome. In addition to adduct formation, AFBO may induce other lesions in DNA, such as specific base damage and/or accerate oxidative DNA damage [14,15]. The toxic potentials of AFB_1_ were commonly evaluated in organs (liver, spleen, etc.), in vivo, or in respective cell models in vitro. Considering the similarities in molecular and cellular pathways to higher eukaryotes, as well as the ease of genetic manipulation [16], *C. elegans* could be a good alternative to in vivo animal models for evaluating genotoxic effects and relevant molecular pathway caused by exposure to AFB_1_ [17,18,19]. In view of above points, in this study, we used *C. elegans* as an in vivo model, and focused on the toxic effects of AFB_1_ impacting on growth and reproduction, as well as DNA damage and germline apoptosis. 

Some commonly used sensitive indicators, growth and reproduction, were used as endpoints for AFB_1_-induced toxicity in *C. elegans*. Our results showed that the body size and egg number were significantly decreased upon the exposure to AFB_1_, especially in the context of NER deficiency (*xpa-1* strain), and these effects seemed to be concentration-dependent. Previously, Yang et al. investigated the toxic effects of AFB_1_ on growth and reproduction of N2 nematodes, and their results indicated that exposure to AFB_1_ would result in significant growth and reproduction inhibition [20]. This is consistent with the current results, which further confirmed that growth and reproduction are sensitive endpoints in evaluating AFB_1_ toxicity in *C. elegan*. Similarly, Leung et al. also found that, even at the concentration of 3 μM, exposure of AFB_1_ could lead to an about 40% size reduction in N2 worms as compared with controls, and the same exposure level of AFB_1_ resulted in a greater growth inhibition (about 60%) in *xpa-1* as compared with N2 [12]. Additionally, Dhanapal et al. found AFB_1_ wielded its toxic effects on hatching and development of embryos of zebrafish in both a time- and concentration-dependent manner [21]. These previous studies suggested that AFB_1_ has a definite toxicity on growth and reproduction in vivo, which was consistent with our findings. Compared to other model organisms, the LC_50_ of AFB_1_ in *C. elegans* was 20.47 mg/L (equivalent to 65.6 µM) [20], considerably higher than mosquito fish (2 µM) and HepG2 cell (1 µM) [22]. In this respect, the *C. elegans* model is less sensitive to the lethality of AFB_1_ than other models.

For the DNA damage assay, a concentration-dependent increase in *C. elegans* nuclear genome lesions was detected following exposure to AFB_1_. It can be noticed that NER-deficient *xpa-1* strain was more susceptible to AFB_1_-induced damage than germ line eficient *glp-1* strain, especially in the lower exposure group (10 μM), with the induced damage rate in *xpa-1* stran being about four times greater than those in *glp-1* strain. This result was consistent with Leung et al., which used a similar approach to measure DNA damage and found detectable lesions after exposures to 30 and 100 µM AFB_1_ in a concentration-dependent manner [12]. It has been well characterized that AFB_1_ can induce DNA damage and lead to carcinogenesis in experimental animals or in human populations. The major AFB_1_-DNA adduct can cause DNA helix-distorting lesions that interfere with base pairing, thereby obstructing transcription and normal replication, in addition to forming apurinic sites via hydrolysis, which have a great potential of developing into mutations if located in transcriptionally-active DNA regions. To maintain genetic integrity, evolution has molded an intricate network of highly conserved DNA repair, one of these system is nucleotide excision repair (NER). NER can deal with the aforementioned helix-distorting lesions and prevent the formation of AP sites [23]. There are several hypotheses as to why DNA damage could be associated with growth inhibition in *C. elegans*. Previous studies have also demonstrated that *C. elegans* DNA repair processes could hinder development, which may keep worms in an early stage with more active DNA repair; one evidence was that available energy would meet the NER demand prior to growth with the presence of DNA damage [13]. The lack of growth also could be due to decreased feeding resulting from a loss in transcriptional competence [24]. The positive correlation between DNA damage and growth inhibition would explain why NER-deficient *xpa-1* show more growth inhibition compared to N2 [24]. The results presented in this study strongly support that the NER system is in charge of repairing the DNA damages caused by AFB_1_. These findings were consistent with previous research conducted in animals or human [25,26].

In *C. elegans*, although the adult somatic cells are invariant, the germ cells are malleable via apoptosis under environmental stress [25]. Upon environmental stresses, germ cell apoptosis can be induced sensitively through the signaling pathways that are genetically distinct from physiological apoptosis. Pioneering research had demonstrated that DNA damage can induce germline apoptosis in *C. elegan*s [26,27,28]. Additionally, much evidence demonstrates that AFB_1_ can induce apoptosis in a variety of cells, such as human liver cells [29], lung cells, bone marrow cells [30], and human lymphocytes [28,31], etc. Considering the known pathway, we decided to investigate whether exposure to AFB_1_ could result in germline apoptosis. By exposing *C. elegans* to graded concentrations of AFB_1_, we found that AFB_1_ induced apoptosis in the germline, especially in higher exposure groups (30 μM and 90 μM). Abnormal germline development, such as induction of germline apoptosis, would definitly lead to reduced egg production. This would explain why exposure to AFB_1_ resulted in the decreased brood size, suggesting that brood size might serve as a sensitive indicator of germline apoptosis.

In response to genotoxic stress, cells activate DNA damage checkpoint pathways that induce DNA repair, cell cycle arrest, or programmed cell death (apoptosis) in order to remove genetically-damaged cells that may harm the organism. The checkpoint signaling cascade is highly conserved among species, and previous study has confirmed that the DNA damage-triggered checkpoint pathway remains effective in *C. elegans* [26]. Apart from checkpoint pathway, recently, some evidence has emerged supporting that the ERK pathway is involved in apoptosis promotion in several cell types under genotoxic or nongenotoxic stresses [32]. The *C. elegans* gene, *egl*-1, encoding the BH3-domain protein, is required for apoptosis under genotoxic stress, whereas *p53*/*cep*-1 induced germ cell death by activating the transcription of the target genes *egl-1* and *ced-1*. In *C. elegans*, *hus-1* and *clk-2* are the checkpoint genes that are required for DNA-damage-induced apoptosis. To address the underlying pathways involved in the germline apoptosis induced by AFB_1_, we used real-time PCR to quantify the expression of the gene invovled in checkpoint pathway and ERK pathway. Our results revealed that the expression of checkpoint genes *hus-1* and *clk-2*, the tumor suppressor gene *p53*/*cep-1*, and the BH3-only domain homolog *egl-1*, were apparently up-regulated upon AFB_1_ exposure when compared to the unexposed control nemotodes, while the members of the ERK signaling pathway (*ksr-1*, *lin45*, and *mek-2*) were not. These data, therefore, suggested that AFB_1_-induced germline apoptosis was mainly associated with the conserved checkpoint pathway and not the ERK pathway.

The concentrations used in this study were selected based on preliminary lethality tests that generated LC_50_ values using the N2 wild-type strain. Recognizing that the absorption, distribution, metabolism, elimination patterns, and mechanisms for chemical toxicants can vary across species despite similar metabolic activation mechanism, *C. elegans* do not metablize AFB_1_ identically to humans. In humans, AFB_1_ is metabolized primarily by the CYP1A2 in liver, while *C. elegans* lacks a CYP1 gene and, instead, is metabolizes AFB_1_ primarily by CYP2 and 3 with different metabolic efficiencies. Additionally, it has been observed that *C. elegans* is considerably less sensitive to lethality of AFB_1_ compared to other model organisms, consequently requiring higher concentration levels for mechanistic exploration. In general toxicology, however, exposure concentrations based on LC_50_ values should reflect the human exposure scenario, given the wide range of the exposure spectra, as well as the known health outcomes in human populations. Regarding relevance to human exposure, if considering the modifying factors (worms to mammals to humans), the concentration-range used in the current study is relevant to moderate and higher levels of human exposure [8,20].

## 4. Conclusions

Our results indicate that exposing *C. elegans* to AFB_1_ caused concentration-dependent growth and reproduction inhibition, nuclear DNA damage, and germline apoptosis. These toxic effects have an underlying interrelationship with each other. Among these effects, we regarded AFB_1_-induced DNA damage as the central hub, as it can lead to growth inhibition and germline apoptosis via checkpoint signaling pathway, and the latter could futher result in inhibition of reproduction. So far, no study has reported DNA damage-induced germline apoptosis and the underlying mechnism of growth or reproduction inhibition cause by exposure of AFB_1_ in *C. elegans*. Our research, therefore, sheds some light onto this field. These results also represent a preliminary stage in developing this genetically tractable organism as a model for assessing AFB_1_ toxicity.

## 5. Materials and Methods

### 5.1. C. elegans Culture

The nematodes used were wild-type N2 strain, and transgenic strains of *glp-1* (germline deficient JK1107) and *xpa-1* (NER-deficient strain RB864), originally obtained from the Caenorhabditis Genetics Center. N2 strain or *xpa-1* mutants were maintained on nematode growth medium (NGM) plates seeded with *Escherichia coli* OP50 at 20 °C, while *glp-1* mutant was typically cultured at 15 °C to maintain normal differentiation [33,34]. By using bleach-sodium hydroxide isolation of eggs, synchronized L1 growth-arrested larvae were obtained by hatching eggs in M9 medium overnight with shaking [35]. These L1 staged nematodes were grown for one day on a NGM plates with OP50 lawn to obtain synchronized L3 staged worm (40 h was needed for *glp-1* mutant). For chemical exposures, L3 staged nematodes were washed off from the agar plate using K medium and centrifuged at 2000× *g* for 10 min [36].

### 5.2. Chemicals Exposures

AFB_1_ (Sigma Chemical Co., St Louis, MO, USA) was dissolved in dimethyl sulfoxide (DMSO; Sigma Chemical Co., St Louis, MO, USA) to prepare stock solutions. Four different concentrations of AFB_1_ (3, 10, 30, and 90 μM) were prepared using serial dilution with DMSO from stock solutions. A preliminary lethality assay (data not shown) has been conducted to determine the exposure concentrations, such that the selected exposure levels would be nonlethal, but could result in reduced movement and growth, which would reflect real-life exposure scenarios in the human population. Synchronized L1 *C. elegans* were treated with the solutions in 12-well sterile tissue culture plates(Corning Inc., Lowell, MA, USA).Each well contained 100 μL of *E. coli* OP50 (optical density of 2.0 at 570 nm), 5 μL of L1 staged worms in M9 buffer (30–40 worms total), 5 μL of AFB_1_ with different concentration or control solution (DMSO), 1 μL of streptomycin (50 mg/mL; Sigma Chemical Co., St Louis, MO, USA), and 890 μL of S medium to bring total volume to 1 mL [37]. AFB_1_ solutions were added to treatment wells at a maximum amount of 0.5% (*v*/*v*) in S medium [35]. DMSO (0.5%) was found not affect nematodes in all tests (data not shown). 

### 5.3. Growth Assay

A growth assay was carried out to investigate toxic effects of AFB_1_ on N2 or *xpa-1* nematodes. Synchronized N2 and *xpa-1* L1 larvae were added to 12-well sterile tissue culture plates, separately. The composition of the cocktail in each well was detailed in the above section (Chemical Exposure). Four wells containing one control were prepared for each toxin concentration. *C. elegans* cohorts were incubated at 20 °C. Each day, for each toxin concentration (including control), 20 worms were taken from one well of the plate. Treated and control *C. elegans* were imaged using a microscope every day for three consecutive days. Using Image J software (Version 1.33, NIH, Bethesda, MD, USA), the body sizes were obtained by measuring flat surface area of nematodes [37]. The growth assay was independently replicated three times. 

### 5.4. Brood Size Assay

Synchronized L1 worms (N2 and *xpa-1* strains) were transferred to NGM plates with OP50 as a food source and incubated at 20 °C for 44 h when the worms reached at the L4 stage. The assay was set up in a 96-well plate in S medium with each well containing a single L4 worm, 0.6 μL of serial dilution AFB_1_ and 10 μL of bacterial culture (OP50) in a total volume of 120 μL [37]. Each AFB_1_ concentration was tested in five wells. When all worms were transferred, the multi-well plates were placed in a constant temperature and humidity incubator, and incubated at 20 °C for three days. After that, the number of progeny in each well was counted. The results for this assay included three independent replicates. 

### 5.5. DNA Damage Assay

To avoid the interference of division of germ line to the measurement of DNA damage, synchronized *glp-1* L3 staged worms were sterilized for 18 h at 25 °C [34]. After that, synchronized *glp-1* and *xpa-1* L3 staged worms were exposed to AFB1 at different concentrations (10, 30, and 90 μM) for 24 h and incubated at 20 °C. After 24 h, worms were picked at a 1 worm per 15 μL lysis buffer (1:15 ratio) as described previously [38]. Six worms were pooled for each biological replicate and five replicates were taken per treatment. DNA damage was determined using a QPCR-based method [39]. The amount of long PCR product (9316 nt) provides a measurement of lesion frequency, whereas the amount of short PCR product (225 nt) provides normalization to the DNA template amount. Lesion calculations were performed as described previously [39]. One major reason to use *glp-1* as a substitute for the N2 strain was that germline proliferation and, therefore, cell division was blocked in *glp-1* while maintained at 25 °C. 

### 5.6. Real-Time PCR 

Synchronized N2 and *xpa-1* L3 staged nematodes were grown at 20 °C and exposed to 10, 30, and 90 μM AFB_1_ for 48 h. Worms were then collected by washing with M9 buffer and frozen in liquid nitrogen. Total RNA was prepared by TRIzol^®^ total RNA Isolation Kit (Life Technology, Carlsbad, CA, USA), according to the manufacturer’s standard protocol. RNA concentrations were determined by the absorbance at 260 nm. The quality of total RNA was estimated based on the ratio of the optical densities from RNA samples measured at 260 and 280 nm. cDNAs were synthesized with random hexamers by using the High Capacity cDNA Reverse Transcription Kit (Life Technology, Carlsbad, CA, USA) according to the manufacturer’s protocol. The real-time PCR primer sequences for the target genes are summarized in Supporting Information Table S1. *act-1* was used as the internal control, and the RNA levels of the genes of interest were normalized to the *act-1* level for comparison. The PCR reaction was initiated at 95 °C for 15 min for denaturation followed by a 40-cycle protocol consisting of 10 s at 95 °C and 30 s at 60 °C. mRNA expression was assessed by quantitative real-time PCR on an ABI 7500 FAST Real-Time PCR System (Applied Biosystems, Foster City, CA, USA) using lightcycle^®^ resolight dye (Roche, Pleasanton, CA, USA) in a 96-well plate. The gene expression data were analyzed using the comparative 2^−ΔΔCT^ method [40]. Triplicates for each sample were included for one single reaction.

### 5.7. Apoptosis Assay 

Apoptotic germ cells were measured by AO staining using a procedure as described previously [41]. Briefly, late-stage L4 nematodes were exposed to different concentrations of AFB_1_ for 48 h, and then were picked from test wells into 24-well tissue culture plates containing 1 mL K-medium with 25 μg/mL AO (Sigma-Aldrich, St. Louis, MO, USA) and incubated for 1 h at 20 °C. To facilitate dye uptake, 30 μL of OP50 broth (optical density of 2.0 at 570 nm) was added to the buffer. Animals were allowed to recover for 1 h on bacterial lawns and then mounted onto agar pads on microscope slides with a drop of 60 μg/mL levamisole in M9 buffer. Apoptotic cells were examined under a fluorescent microscopy (Olympus America Inc. Center Valley, PA, USA). At least 20 worms were measured for each treatment. The apoptotic cells appeared yellow or yellow-orange, which represented increased DNA fragmentation, whereas intact cells were uniformly green [42]. In most conditions, only the posterior arm of the gonad could be scored because the autofluorescence of the intestine always shaded the gonad arm near the pharynx. 

### 5.8. Statistical Analysis

All data were presented as means of at least three independent experiments (triplicated within independent samples) with standard deviation (SD) or standard error (SEM). The median effective concentration (EC_50_, concentration producing a 50% reduction in body size or offsprings compared to control) with 95% confidence intervals (CI), were calculated using logistic regression. Response variables that were not normally distributed were transformed logarithmically to improve normality. Williams’ test was carried out to test the concentration-related trend and significance [43]. Dunnett’s test was used to assess the significance of pairwise comparisons between exposed and control groups. Data analysis were performed using the R package nparcomp (R Core Team, version 3.3.2, Vienna, Austraia, www.r-project.org), and a *p*-value of 0.05 or less was considered to be statistically significant.

## Figures and Tables

**Table 1:** The median effective concentrations (EC_50_, µM) of AFB_1_ exposure on growth inhibition.

Time (h)	N2 (95% CI)	*xpa-1* (95% CI)
24	7.87 (6.93–8.93)	5.02 (4.38–5.76)
48	6.36 (5.91–6.86)	4.25 (3.92–4.62)
72	7.40 (6.79–8.01)	3.54 (3.37–3.71)

## Supplementary Materials

The following are available online at www.mdpi.com/2072-6651/9/1/9/s1, Table S1: Summary of the real-time PCR primers of the target genes in this study.
