# Supplementary material for: Aflatoxin B1-Induced Developmental and DNA Damage in Caenorhabditis elegans
Source: Toxins (Basel). 2016 Dec 26;9(1):9. doi: 10.3390/toxins9010009 (PMC5308242; doi:10.3390/toxins9010009)
Supplement: Supplementary file 1 [file toxins-09-00009-s001.pdf]

Supplementary Materials: Aflatoxin B_1-Induced Developmental and DNA Damage in *Caenorhabditis elegans*

Wei-Hong Feng, Kathy S. Xue, Lili Tang, Phillip L. Williams and Jia-Sheng Wang

Table S1. Summary of the real-time PCR primers of the target genes in this study.

Gene Symbol	Forward primer	Reverse primer	Product length
act-1	TCACCGCTCTTGCCCCATCA	TTGTGCATTTAGAAGCACTTGCGGT	187
egl-1	TCAATTTGCCGACGACTCGGG	TCTGTCGGAAGCATGGGCCG	139
hus-1	GTTCTGCCCGGCGGACACTG	TGTGTCCAATTGACGGCCTGGA	178
clk-2	GCGAGAGCCACCAAGAGCCG	GGCAATGATGCAGGCAGTCCGT	274
cep-1	TGTATCCAGGCGCAGTT	TTGTTTGATGTATGCGTGGAG	152
ksr-1	ACAATCAGAGGTCCTAATGC	GGTGTAAACTTTGACTGCG	159
lin-45	ATTCTTCGGAACTGTTGCTA	ATTTCTGGTTCTCGAACCC	150
mek-2	GATTGAACTGGCTGATAGTCT	AAATCCAATCACTTCGTCAGTAA	151
